# Environmental Factors Related to Fungal Wound Contamination after Combat Trauma in Afghanistan, 2009–2011

**DOI:** 10.3201/eid2110.141759

**Published:** 2015-10

**Authors:** David R. Tribble, Carlos J. Rodriguez, Amy C. Weintrob, Faraz Shaikh, Deepak Aggarwal, M. Leigh Carson, Clinton K. Murray, Penny Masuoka, on behalf of the Infectious Disease Clinical Research Program Trauma Infectious Disease Outcomes Study Group

**Affiliations:** Uniformed Services University of the Health Sciences, Bethesda, Maryland, USA (D.R. Tribble, A.C. Weintrob, F. Shaikh, D. Aggarwal, M.L. Carson, P. Masuoka);; Walter Reed National Military Medical Center, Bethesda (C.J. Rodriguez, A.C. Weintrob);; Henry M. Jackson Foundation for the Advancement of Military Medicine, Inc., Bethesda (A.C. Weintrob, F. Shaikh, D. Aggarwal, M.L. Carson, P. Masuoka);; San Antonio Military Medical Center, Joint Base San Antonio, Fort Sam Houston, Texas, USA (C.K. Murray)

**Keywords:** military medicine, mucormycosis, zygomycosis, wound infection, trauma, Afghanistan, fungi, Aspergillus species, mold

## Abstract

During the recent war in Afghanistan (2001–2014), invasive fungal wound infections (IFIs) among US combat casualties were associated with risk factors related to the mechanism and pattern of injury. Although previous studies recognized that IFI patients primarily sustained injuries in southern Afghanistan, environmental data were not examined. We compared environmental conditions of this region with those of an area in eastern Afghanistan that was not associated with observed IFIs after injury. A larger proportion of personnel injured in the south (61%) grew mold from wound cultures than those injured in the east (20%). In a multivariable analysis, the southern location, characterized by lower elevation, warmer temperatures, and greater isothermality, was independently associated with mold contamination of wounds. These environmental characteristics, along with known risk factors related to injury characteristics, may be useful in modeling the risk for IFIs after traumatic injury in other regions.

## Abstract 2

Environmental characteristics, along with known risk factors, may help predict likelihood of mold contamination after injury.

Trauma-related invasive fungal infections (IFIs) generally develop as a complication after a penetrating wound has been inoculated with fungal spores. Although trauma-related IFIs are not as common as bacterial infections, they are characterized by substantial morbidity (e.g., limb amputations) and mortality rates as high as 38% in civilian populations (*1*–*7*). Trauma-related IFIs have been reported in persons who sustained injuries in agricultural/industrial accidents, natural disasters, and combat (*2*–*12*).

One of the largest reported series of trauma-related IFIs occurred among military personnel who sustained combat-related injuries in Afghanistan (77 case-patients and an overall incidence rate of 6.8%) (*13*,*14*). In a multivariable analysis, the following risk factors were independently associated with the development of IFIs: blast injuries sustained while person was on foot patrol (dismounted), traumatic above-knee amputations, and super-massive (>20 units) transfusions of packed red blood cells during the first 24 hours after injury (*14*).

Although location was not included in the risk factor analysis in the previous study, clinicians have recognized that military personnel with IFIs predominantly sustained injuries in the southern provinces of Afghanistan (i.e., Helmand and Kandahar) (*6*,*14*). As with other regional infectious diseases, climate and environmental conditions may be key factors. Because of the high morbidity and mortality resulting from trauma-related IFIs, specifically identifying any potential factors associated with exposure is critical to mitigating risk by supporting timelier diagnosis and treatment. Therefore, our objectives were to assess traumatic wound mold contamination in regards to environmental conditions unique to injury circumstances for individual patients and also to investigate geographic and environmental factors associated with regional grouping of cases.

## Methods

### Study Population

The study population included 1,133 US military personnel who sustained combat-related injuries in Afghanistan from June 1, 2009, through August 31, 2011. After medical evacuation to Landstuhl Regional Medical Center in Landstuhl, Germany, the patients were transferred to 1 of 3 participating military hospitals in the United States: Walter Reed Army Medical Center (Washington, DC, USA), National Naval Medical Center (Bethesda, MD, USA), and Brooke Army Medical Center (San Antonio, TX, USA). Data from these patients were collected as part of a longitudinal, prospective cohort study which analyzed infectious complications among military personnel with deployment-related trauma, the US Department of Defense–Department of Veterans Affairs, Trauma Infectious Disease Outcomes Study (*15*). Characteristics and injury circumstances for patients who met criteria for inclusion in the analysis (i.e., injured during deployment, >18 years of age, evacuated from theater to Landstuhl Regional Medical Center, and transferred to a participating hospital in the United States) were collected from the Department of Defense Trauma Registry (*16*). The study received approval from the Infectious Disease Institutional Review Board of the Uniformed Services University of the Health Sciences (Bethesda, MD, USA).

### Case-Patient and Control-Patient Identification

In a prior nested case–control study within a cohort that examined risk factors for the development of an IFI after a combat-related injury, we identified 76 IFI case-patients from 1,133 wounded personnel using established IFI case definitions and matched them with 150 control-patients by injury severity score (±10) and date of injury (±3 months) (*14*). For the nested case–control study described here, we retrieved grid coordinates for the location of injury for patients who were part of the original case–control study from the US Department of Defense Joint Trauma Analysis and Prevention of Injury in Combat program (Fort Detrick, MD, USA) and imported the data to ArcGIS, a geographic information system (GIS) software package (http://www.esri.com). Because grid coordinates were not available for all patients included in the original analysis, the analysis herein incorporates data from a subset of the cohort (71 IFI case-patients and 101 control-patients). Although the original analysis involved matching, the analysis described here was an unmatched case–control analysis.

**Figure 1:**
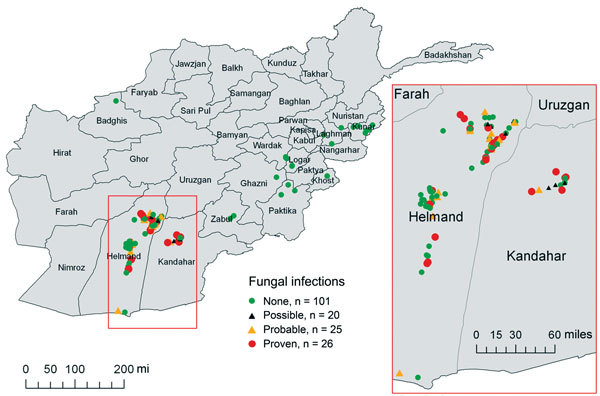
Geographic distribution of 71 case-patients with invasive fungal wound infections and 101 matched control-patients. Afghanistan, 2009–2011. Inset shows a detailed view of southern Afghanistan region where most cases originated. The IFI case-patients are classified according to established definitions (*13*). A proven IFI is confirmed by angioinvasive fungal elements on histopathologic examination. A probable IFI had fungal elements identified on histopathologic examination without angioinvasion. A possible IFI had wound tissue grow mold; however, histopathologic features were either negative for fungal elements or a specimen was not sent for evaluation. In addition, to be identified as an IFI, the wound must demonstrate recurrent necrosis after at least 2 surgical débridements. Because injuries frequently occurred in close proximity, some points overlay other points.

As previously stated, the observed IFI cases have been identified to cluster in the southern region of Afghanistan (Figure 1) (*6*,*17*). The subset of 172 patients with available grid coordinates were classified on the basis of the presence of mold wound contamination, whether or not the patient’s condition progressed to an IFI. This classification is based on the wound microbiology results obtained at either Landstuhl Regional Medical Center or at hospitals in the United States. Specifically, the mold contamination group included patients with wound cultures that grew mold. If wound cultures did not grow mold, but the patient’s wound had histopathologic features or angioinvasion that met the case definition of an IFI (*13*), the patient was included in the mold contamination group. By these criteria, all 71 IFI case-patients were identified as having fungal infections. Patients who had no mold growth and did not meet IFI case definitions were included in the noncontaminated group.

For our analysis, we selected 2 combat zones in Afghanistan (southern and eastern regions) based on casualty rates and compared the regions using environmental variables. The southern region included Helmand and Kandahar Provinces and the eastern region consisted of the area east and north of Zabul Province (Figure 1). Five patients injured outside the southern and eastern regions were excluded from the analysis.

### Environmental Data

Two digital datasets were used for a visual comparison with injury locations. Land cover data, originally developed by the Food and Agriculture Organization using 1992 satellite data, were downloaded from MapCruzin (http://www.mapcruzin.com). River data, originally part of the Digital Chart of the World dataset, were downloaded from DIVA-GIS (http://www.diva-gis.org.

For the statistical analysis and modeling, we obtained several raster environmental datasets and imported them to ArcGIS version 10.2; using the “Extract Values to Points” option, the values of the pixels at injury locations were copied from each environmental layer and recorded in a table for further analysis. We obtained 1-km bioclimatic raster data from WorldClim (http://www.worldclim.org); variables included datasets such as precipitation of the wettest month and minimum temperature of the coldest month of the year. These variables are derived from averages of 50 years of temperature and precipitation measurements (*18*). Elevation data, measured by the Shuttle Radar Topography Mission, were reformatted to the same resolution as the other bioclimatic variables and downloaded from WorldClim. Normalized Difference Vegetation Index (NDVI) data were obtained from National Aeronautics and Space Administration’s Level 1 and Atmosphere Archive Distribution System website (http://ladsweb.nascom.nasa.gov). The NDVI product used was 1-km monthly data collected from the Terra satellite (product no. MOD13A3). NDVI is a measure of the amount of healthy green vegetation on the ground based on the amount of red and near-infrared reflectance measured by satellite. Low NDVI values are associated with water, snow, sand, rock, and dead vegetation, whereas high values represent healthy green vegetation such as forests and grasslands.

### Ecologic Niche Modeling

An ecologic niche model of Afghanistan was produced in MaxEnt 3.3.3k (http://www.cs.princeton.edu/~schapire/maxent/) (*19*) by using the bioclimatic variables, elevation data, and all locations where wounds were contaminated with mold. The model was projected onto Iraq to determine environmentally similar locations where fungal infections might have been expected in wounds during the Gulf War. MaxEnt, which uses a maximum entropy algorithm, uses a set of raster environmental layers (e.g., temperature and elevation) and information on species presence locations, determines the environmental requirements of the species on the basis of environmental conditions at the presence locations, and produces output maps that predict the probability of presence of a species. MaxEnt uses presence-only data rather than presence/absence data and has been shown to be a high-performing model-building program (*20*) and excels in using small numbers of occurrence points (*21*). 

Twenty-five percent of the occurrence points were used for testing the model accuracy (testing points), and the remaining 75% were used for building the model (training points); training and testing points are randomly selected by MaxEnt. As a measure of the accuracy of the model, MaxEnt calculates the area under the curve (AUC) of the receiver operating characteristic of both the training and testing points (*19*,*22*,*23*). To provide estimates of the potential contribution of environmental variables to the model, MaxEnt uses a jackknife test in which the model is run multiple times, with only 1 environmental layer used at a time and then all but 1 variable used, to determine the training gain of each variable in each model (*24*).

### Statistical Analysis

The Wilcoxon rank-sum test was used to compare the geographic characteristics between the 2 regions in Afghanistan. In addition, an unconditional logistic regression model was used to analyze the association between potential environmental risk factors and mold contamination of wounds in a univariable and multivariable analysis. Backward elimination was used to determine which factors that were significant in the univariable analysis remained in the final multivariable model. A correlation analysis was also conducted to examine the relationship between the environmental factors. Statistical analysis was conducted by using SAS version 9.3 (SAS Institute, Inc., Cary, NC, USA). Significance level for all statistical tests was 0.05.

## Results

### Study Population and Injury Characteristics

**Figure 2:**
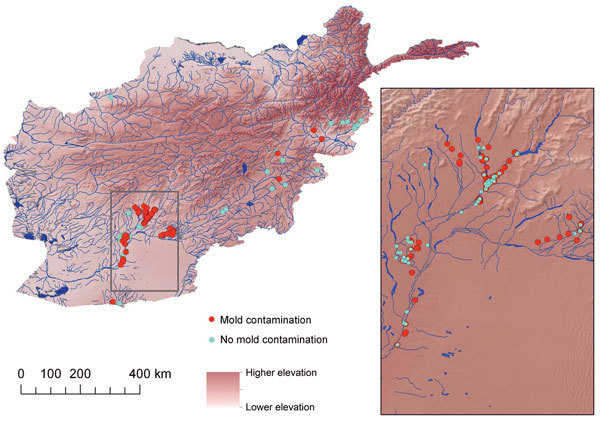
Geographic distribution of military personnel with wounds contaminated by mold (n = 101) and control-patients (n = 66), Afghanistan, 2009–2011. Inset shows detail view of southern Afghanistan region where most cases originated. The mold contaminated group includes 7 patients for whom cultures did not show mold growth, but were diagnosed with invasive fungal wound infections (IFIs) on the basis of histopathologic examination. Five patients with injuries sustained outside the study regions were excluded from the analysis (including 1 patient with IFI), but remain visible on the map (i.e., 1 point in western region, 2 in the southernmost point of Helmand Province below the box indicating the southern analysis region, and 2 in Zabul Province between the southern and eastern regions). Because injuries frequently occurred in close proximity, some points overlay other points. The mold contamination points are on top on the overview map and on the bottom in the enlarged inset. Higher elevation is indicated by darker brown.

The location of injury and IFI status of the 172 patients used as the basis for the mold wound contamination analysis are shown in Figure 1. After 167 patients were classified by mold contamination status (101 patients with mold contamination and 66 without), 147 military personnel injured in the southern region of Afghanistan (area favorable to mold wound contamination) and 20 injured in eastern Afghanistan (less frequent mold wound contamination) were included in the analysis (Figure 2). As previously stated, 5 patients (including 1 IFI case-patient) were excluded because the injury occurred outside of the study regions. Cultures from 7 patients in the mold contamination group included in the analysis did not have mold growth, but the patients received a diagnosis of IFIs on the basis of histopathologic examination. Notably, all remaining IFI case-patients sustained injuries in the southern region, whereas both regions contained patients with mold wound contamination.

Although the injury mechanism was predominantly blast (e.g., improvised explosive device, rocket-propelled grenade, and grenade) for military personnel injured in both regions, more service members were injured while dismounted in the southern region of Afghanistan (95% vs. 60%; p<0.001). In addition, patients injured in southern Afghanistan also had significantly higher injury severity scores (median 21 vs. 17.5; p = 0.033) and a more above-knee amputations (46% vs. 5%; p<0.001). Correspondingly, southern Afghanistan also had a significantly higher proportion (50% vs. 5%; p = 0.004) of wounded military personnel that required super-massive transfusions (>20 units) of packed red blood cell within 24 hours following injury.

### Factors Associated with Mold Wound Contamination 

The spatial distribution of patients included in the analysis, based on the presence or absence of mold wound contamination, is displayed in Figure 2. Overall, mold was recovered more frequently from wounds of patients injured in southern Afghanistan (61% vs. 20%; p<0.001). When we included patients who did not have mold growth but did have IFIs that were diagnosed on the basis of histopathologic features, 66% of those injured in southern Afghanistan had mold contamination. The variables of region (southern vs. eastern), NDVI, elevation, annual precipitation, annual mean temperature, and annual range of temperature were examined for association with wound mold contamination on a per individual basis (Table 1). Annual precipitation was significantly associated with mold contamination (p = 0.01) on univariable analysis but was not included in the final multivariable model because of its high correlation with temperature variables. Injuries sustained in southern Afghanistan were significantly more likely to be contaminated with mold (p = 0.001), and region was the only factor retained as a statistically significant independent predictor in the final multivariable model (p = 0.001).

**Table 1:** Environmental risk factor analysis for mold contamination of wounds, Afghanistan, 2009–2011*

Environmental factor	Univariable OR (95% CI)	p value†	Multivariable OR (95% CI)	p value‡
NDVI§	0.93 (0.49–1.77)	0.89	1.16 (0.58–2.33)	0.68
Elevation	1.00 (0.99–1.00)	0.11	1.00 (1.00–1.01)	0.61
Annual precipitation¶	0.99 (0.99–1.00)	0.01	−	−
Annual mean temperature	1.09 (0.95–1.24)	0.22	0.84 (0.42–1.67)	0.61
Temperature annual range	1.19 (0.97–1.47)	0.10	0.74 (0.50–1.09)	0.12
Regional area				
East	Reference		Reference	
South	7.76 (2.46–24.4)	0.001	129.9 (7.71–>999)#	0.001
*OR, odds ratio; NDVI, normalized difference vegetation index. †For univariable analysis. ‡For multivariable analysis. §The NDVI uses a mathematical formula to quantify the density of healthy green vegetation in a region (*25**,**26*). The NDVI values were normalized by taking the natural log of the index. ¶Because of its high correlation with the temperature variables, annual precipitation was not included in the final multivariable model. #The wide CI is due to correlation between the regional and the environmental variables.				

### Regional Environmental Characteristics

Because mold-contaminated wounds were more frequent in southern Afghanistan than in eastern Afghanistan (Figure 2), we compared the environmental factors. The elevation of the southern Afghanistan study zone was significantly lower (p = 0.001) than that of the eastern region (Table 2). The regions also exhibited differences in temperature. Southern Afghanistan was generally warmer with the annual mean temperature (p<0.001), mean diurnal range (p<0.001), and isothermality (p<0.001) higher than the eastern region. We found no statistically significant difference in temperature seasonality, as expressed by the SD of the weekly mean temperatures and the annual temperature range between the 2 regions. Although southern Afghanistan had a lower amount of annual precipitation (p<0.001) than the eastern region, the variable did not differentiate between rain and snow. Vegetative cover, as categorized by the NDVI, was comparable between the 2 regions.

**Table 2:** Environmental characteristics of 2 regions in Afghanistan associated with combat-related trauma, June 2009–August 2011*

Characteristic	Southern Afghanistan, median (IQR)	Eastern Afghanistan, median (IQR)	p value†
NDVI‡	7.64 (7.23–7.95)	7.69 (7.52–8.00)	0.384
Elevation, m	902 (859–946)	1,670 (1,050–2,117)	<0.001
Isothermality§	4.2 (4.1–4.2)	3.4 (3.4–3.5)	<0.001
Temperature, °C			
Annual mean	18.8 (18.6–19.5)	13.9 (10.3–18.3)	<0.001
Seasonality¶	880.7 (877.9–884)	861.7 (853.6–964.0)	0.471
Mean diurnal range	17.4 (17.3–17.5)	13.5 (12.7–15.0)	<0.001
Maximum of warmest month	40.8 (40.5–41.3)	33.4 (31.5–36.6)	<0.001
Minimum of coldest month	−0.6 (−0.8 to 0)	−3.8 (−12 to 0.5)	0.194
Annual range	41.4 (41.3–41.5)	36.5 (35.8–43.3)	0.138
Mean of wettest quarter	9.6 (9.2–10.3)	8.1 (4.8–12.5)	0.681
Mean of driest quarter	30.1 (29.6–30.8)	19.3 (17.8–21.3)	<0.001
Mean of warmest quarter	30.2 (29.9–30.8)	25.3 (22.5–29.3)	<0.001
Mean of coldest quarter	7.3 (7.0–7.9)	2.7 (−2.9 to 7.1)	<0.001
Precipitation, mL			
Annual	145 (130–154)	443 (2,795–546)	<0.001
Wettest month	39 (35–40)	89 (64–123)	<0.001
Driest month	0	6 (0–12.5)	<0.001
Seasonality#	110 (108–115)	82 (73–90)	<0.001
Wettest quarter	103 (92–109)	210 (168–307)	<0.001
Driest quarter	0	28 (8–51)	<0.001
Warmest quarter	0	46 (10–66)	<0.001
Coldest quarter	90 (80–95)	125 (113–151)	<0.001
*Environmental characteristics were obtained in relation to grid coordinates on a per-individual basis: southern Afghanistan region (147 patients) and eastern Afghanistan region (20 patients). IQR, interquartile range; NDVI, normalized difference vegetation index. †p values were calculated using Wilcoxon rank-sum test. The regional variables were treated as dependent, whereas the environmental characteristic was independent. ‡NDVI uses a mathematical formula to quantify the density of healthy green vegetation in a region. Higher values indicate healthy green vegetation (e.g., forests), while lower values are associated with water, snow, sand, rock, and dead vegetation (*25**,**26*). The NDVI values were normalized by taking the natural log of the index. §Mean diurnal range divided by temperature annual range and then multiplied by 100. ¶SD of weekly mean temperature multiplied by 100. #Coefficient of variation.			

**Figure 3:**
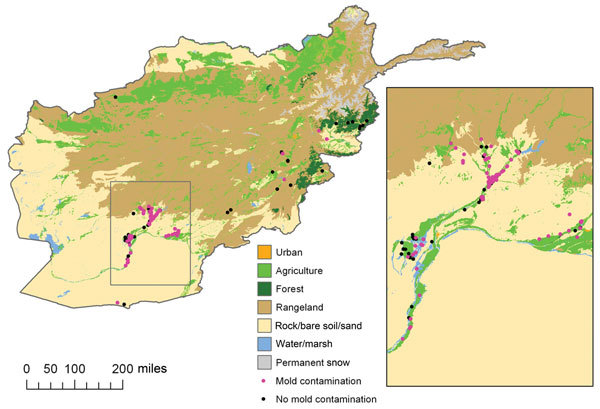
Geographic features of southern and eastern Afghanistan study zones, 2009–2011. Inset shows detail view of southern Afghanistan region where most cases originated. Because injuries frequently occurred in close proximity, some points overlay other points. The mold contamination points are on top.

The southern Afghanistan study zone included the provinces of Kandahar and Helmand. In general, the region is arid and characterized by flat grasslands or rangelands with areas of agriculture. A portion of control-patients sustained injuries in the agricultural areas of southern Afghanistan, but most military personnel injured in this region had mold-contaminated wounds (Figures 2, 3). 

**Figure 4:**
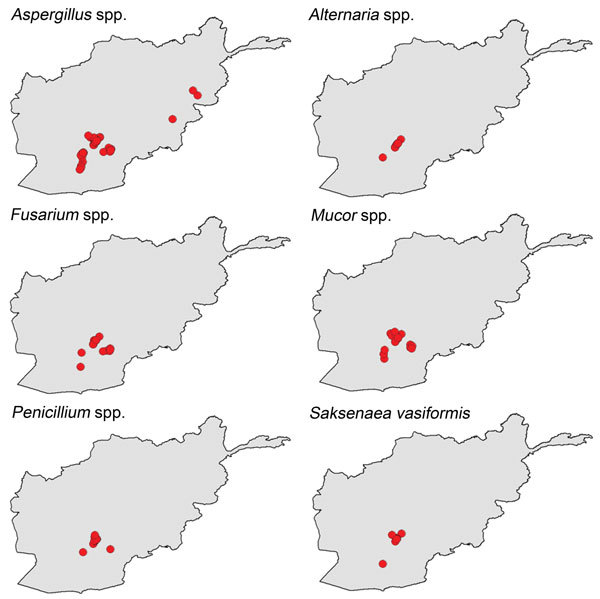
Geographic distribution of specific molds isolated from wounds sustained by military personnel in Afghanistan, 2009–2011.

The eastern Afghanistan study zone included 9 provinces northeast of the southern study zone. The terrain in eastern Afghanistan is largely mountainous with small forested and agricultural areas. As with southern Afghanistan, the 4 patients with mold-contaminated wounds sustained injuries in the vicinity of agricultural zones (Figures 2, 3). It is notable that the species of molds that grew in patients injured in southern Afghanistan are known to be pathogenic, such as the order Mucorales and *Aspergillus* spp. (Figure 4).

### Ecologic Niche Modeling

A total of 61 points were used to build the model (training points), and 20 points were withheld to test the Afghanistan model. The Afghanistan model had a high AUC value (0.963), indicating that the model is predicting better than a random model (0.5) and in the very high (>0.9) accuracy range (*22*). In the jackknife test, variables of mean diurnal range, isothermality, and annual precipitation had the highest training gains, which indicated that they had the greatest predictive abilities.

## Discussion

During the recent military conflicts, combat-related IFIs have largely occurred in military personnel who sustained dismounted blast injuries in southern Afghanistan. Although risk factors related to the severity and circumstances of injury have been analyzed (*14*), the effect on disease of the region’s climate and environmental conditions has not been previously considered. We compared environmental data from a region in Afghanistan associated with a high risk of mold contamination of wounds (mold was recovered from 61% of the high-risk patients in this cohort) to a region where mold was infrequently reported (20%) among combat wounds. Results of the multivariable analysis confirmed that injuries (on an individual basis) sustained in the southern region of Afghanistan were more likely to be contaminated with mold (odds ratio [OR] 129.9, 95% CI 7.71 to >999), which corroborates clinical observations. Because case-patients with mold contamination were grouped in a similar manner to the IFI case-patients (Figures 1, 2), we believe that these data will be applicable to potential future IFI outbreaks related to combat situations.

Along with the environmental characteristics of a region, circumstances of injury play a major role in creating the potential for mold contamination of wounds and subsequent likelihood of progression to an IFI. Notably, the proportion of military personnel in southern Afghanistan with IFI risk factors (i.e., dismounted blast injuries, massive blood transfusions, and above-knee amputations) was significantly greater than that of personnel in the eastern region. In particular, more personnel in southern Afghanistan were dismounted at the time of injury, sustained a greater number of traumatic above-the-knee amputations, and required more super-massive packed red blood cell transfusions.

In addition, our data indicated that the southern region of Afghanistan was generally warmer than the eastern region with greater isothermality, which is consistent with conditions favorable for mold growth. In particular, high humidity and temperatures of ≈27°C have been reported as the optimal conditions for growth of mold from the order Mucorales i (*27*). Similar observations have been reported for *Aspergillus* spp., with optimal temperatures ranging from 25°C to 40°C (*27*). Although precipitation was not retained in the multivariable analysis, it was significantly associated with mold contamination in the univariable analysis (Table 1). Nonetheless, annual precipitation was significantly lower in southern Afghanistan, an arid environment (Table 2). Because moisture is necessary for mold growth, we believe that rivers and agricultural irrigation are playing a large role in southern Afghanistan. Similar NDVI rates in the southern and eastern regions, in spite of differences in annual precipitation rates, lend support to this idea. Another explanation is that eastern Afghanistan has a significantly higher elevation and lower temperatures. Thus, a proportion of the precipitation may have been snow, which is not conducive to mold growth. Future attempts might improve the model by incorporating additional data reflecting ground moisture, runoff, and/or irrigation presence.

Approximately 3,300 species of soil fungi have been identified worldwide (*27*). Analyses of the military personnel with IFIs found that the predominant fungi involved were Mucorales and *Aspergillus* spp. (*6*,*13*). Species in the order Mucorales are commonly found on decaying organic matter, crop debris, compost piles, animal excreta, and agricultural/forest soils (*27*). *Aspergillus* spp. are another fungi frequently found worldwide in agricultural, forest, grassland, wetland, and desert soils (*28*). Although fungal species are found worldwide, local variations in the incidence of the individual species have been reported in soils and plants due to differences in such factors as temperature, humidity, and host plant species (*29*–*33*). Cereal crops (e.g., wheat, rice, maize, and barley), which are frequently associated with growth of Mucorales and *Aspergillus* spp. in the soil (*28*,*34*), are predominant in Helmand Province, but production is often affected by resource variability due to drought and flooding. Moreover, agriculture in Afghanistan also includes the illegal growth of poppies for opium production (*35*), with a reported 806 square miles planted across Afghanistan in 2013 (*36*). Notably, provinces in southern Afghanistan (i.e., Helmand and Kandahar) contribute 73% to the overall growth of poppies in Afghanistan (*35*). Agricultural changes such as these in provinces where military personnel are injured may result in differing exposures to soil molds and may explain the geographic variation of the case-patients.

**Figure 5:**
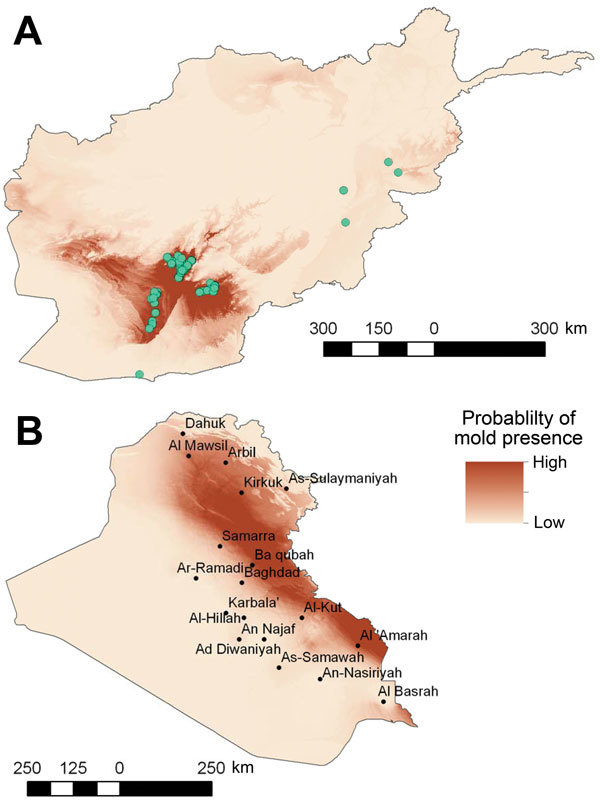
Results of ecologic niche modeling in Afghanistan, 2009–2011 (A), and projection of findings onto Iraq (B). Darker red indicates areas estimated to have higher probability of mold presence based on the environmental conditions of mold contamination locations in Afghanistan (green circles).

Despite statistically significant differences in the environmental characteristics of the 2 regions, we are not able to definitively conclude that the environmental conditions were directly associated with increased risk for mold wound contamination among wounded military personnel. Nevertheless, one can reasonably assume that the environmental conditions made mold wound growth more likely in conjunction with the specific scenario of mechanism and pattern of injury. Thus, environmental data obtained in our analysis may be extrapolated and used in niche modeling in an effort to speculate on the likelihood of IFIs in different regions with similar circumstances of combat-related injuries. As a theoretical example, we developed an ecologic niche model for Afghanistan (Figure 5) using MaxEnt (*19*) and projected the model on Iraq. The Iraq model shows regions where the environmental conditions are similar to the environmental conditions in southern Afghanistan where mold wound contamination frequently occurred. A very low incidence of combat-related IFI was reported among combat casualties in Iraq (*12*), possibly due to less frequent dismounted blast injuries, However, we did not have the grid coordinates where injuries were sustained to evaluate whether the locations were consistent with our predictive map. Although we cannot draw conclusions about where personnel were injured or how accurate the model is for Iraq, military operations did occur in the areas indicated by the predictive map (Figure 5).

A potential limitation of our analysis is the lack of environmental mold sampling; however, whether this would be contributory or as valuable as wound contamination sampling is not clear. From a biogeographic perspective, sampling wounds for mold contamination is not the ideal sampling method for determining where mold is abundant in the environment. For this study, we were unable to obtain soil and plant samples to confirm or disprove the idea that certain regions are more likely to support the development of IFIs after traumatic injury. Although impractical in a war zone and expensive to implement, given adequate mold samples over a wide geographic region and range of environmental factors, the ecologic niche model developed in this article could be improved and may ultimately be a useful tool. For example, mold samples from soils and vegetation have proven useful in agricultural research in determining factors associated with poor crop health (*30*–*32*). Thus, similar sampling may prove useful in predicting geographic areas with a higher likelihood for mold wound contamination, allowing clinicians to have a heightened awareness of the risk for IFI among patients with severe traumatic injuries. This awareness will contribute to earlier diagnosis and more timely treatment of the disease.

Overall, our data indicate that the environmental conditions in southern Afghanistan were favorable to mold growth, particularly when a specific mechanism and pattern of injury occurred. We also believe that the specific environmental characteristics may be applied to predictive modeling that would be useful during future military conflicts in situations in which the injury mechanism and pattern match known IFI risk factors.
